# Potential predictors of severe cardiovascular involvement in Marfan syndrome: the emphasized role of genotype–phenotype correlations in improving risk stratification—a literature review

**DOI:** 10.1186/s13023-021-01882-6

**Published:** 2021-05-31

**Authors:** Roland Stengl, Bence Ágg, Miklós Pólos, Gábor Mátyás, Gábor Szabó, Béla Merkely, Tamás Radovits, Zoltán Szabolcs, Kálmán Benke

**Affiliations:** 1 Heart and Vascular Center, Semmelweis University, Városmajor u. 68, Budapest, 1122 Hungary; 2 Hungarian Marfan Foundation, Városmajor u. 68, Budapest, 1122 Hungary; 3 Department of Pharmacology and Pharmacotherapy, Semmelweis University, Üllői út 26, Budapest, 1085 Hungary; 4 Center for Cardiovascular Genetics and Gene Diagnostics, Foundation for People With Rare Diseases, Wagistrasse 25, 8952 CH-Schlieren-Zurich, Switzerland; 5 Department of Cardiac Surgery, University of Halle, Halle, Germany

**Keywords:** Marfan syndrome, Genotype–phenotype correlations, Predictors, Prophylactic surgery

## Abstract

**Background:**

Marfan syndrome (MFS) is a genetically determined systemic connective tissue disorder, caused by a mutation in the *FBN1* gene. In MFS mainly the cardiovascular, musculoskeletal and ocular systems are affected. The most dangerous manifestation of MFS is aortic dissection, which needs to be prevented by a prophylactic aortic root replacement.

**Main body:**

The indication criteria for the prophylactic procedure is currently based on aortic diameter, however aortic dissections below the threshold defined in the guidelines have been reported, highlighting the need for a more accurate risk stratification system to predict the occurrence of aortic complications. The aim of this review is to present the current knowledge on the possible predictors of severe cardiovascular manifestations in MFS patients, demonstrating the wide range of molecular and radiological differences between people with MFS and healthy individuals, and more importantly between MFS patients with and without advanced aortic manifestations. These differences originating from the underlying common molecular pathological processes can be assessed by laboratory (e.g. genetic testing) and imaging techniques to serve as biomarkers of severe aortic involvement. In this review we paid special attention to the rapidly expanding field of genotype–phenotype correlations for aortic features as by collecting and presenting the ever growing number of correlations, future perspectives for risk stratification can be outlined.

**Conclusions:**

Data on promising biomarkers of severe aortic complications of MFS have been accumulating steadily. However, more unifying studies are required to further evaluate the applicability of the discussed predictors with the aim of improving the risk stratification and therefore the life expectancy and quality of life of MFS patients.

## Background

Marfan syndrome (MFS) is a systemic connective tissue disorder, affecting approximately 1 in 3000–5000 people [1]. The main clinical features are presented in the cardiovascular, musculoskeletal and ocular systems [2, 3]. MFS is inherited in an autosomal dominant manner, but around 25% of the mutations are de novo. No predilection exists regarding race, ethnicity or gender, and it is important to highlight the considerable variability of the symptoms between and within families [4, 5]. The diagnosis of the disease is based on the revised Ghent nosology, in which aortic involvement, ectopia lentis and genetic background are emphasized more than in previous guidelines [6]. The management of MFS patients require a multidisciplinary approach, and apart from the physical symptoms, psychological factors need to be taken into consideration as they can be quite different in people with MFS compared to individuals without the disease [7].

## Genetic background and pathomechanism

MFS is caused by a mutation of the *FBN1* gene located on the long arm of chromosome 15 [3]. To date more than 3000 genetic variants of the *FBN1* gene have been reported [8] (HGMD Professional 2020.3). The most common mutation type in MFS is missense, of which the ones involving a cysteine amino acid appear most frequently. Other variants include nonsense and frameshift mutations leading to premature termination codon (PTC) as well as in-frame deletions/insertions and splicing-affecting intronic mutations [9]. Copy number variations (CNVs > 50 bp) and other structural variants can also be responsible for the disease [10, 11]. Cysteine, of which more than 360 can be found in fibrillin-1, has a particularly important role in the tertiary structure of this protein through the formation of disulfide bridges [9]. Fibrillin-1 contains 46–47 epidermal growth factor (EGF) domains, 42–43 of which are calcium binding. Each EGF domains have six highly conserved cysteine residues that are involved in disulfide bond formation [12]. Furthermore, there are 7 TB (TGF-β-binding protein-like) domains that contain 8 cysteine each and further 2 hybrid domains [13].

Fibrillin-1 is a key component in the extracellular matrix. Fibrillin-1 molecules build up microfibrils that either connect with elastin to form elastic fibres, which is the case in the walls of elastic arteries, or fulfill their structural function without elastin, for which ciliary zonules in the eye are an example [13]. Fibrillin-1 also has a regulatory role in transforming growth factor-β (TGF-β) signaling. TGF-β is synthesized as an inactive precursor and it contains a prodomain called latency associated peptide (LAP), and they together form the so called small latent complex (SLC). The LAP covalently binds to a latent TGF-β binding protein (LTBP) forming the large latent complex (LLC), which is connected to fibrillin-1 through the LTBP, and this way TGF-β is sequestered in the extracellular matrix [13, 14]. Therefore, in case of fibrillin-1 disruption, TGF-β sequestration fails resulting in increased plasma TGF-β level. Excessive TGF-β signaling is an important aspect of the pathogenesis of MFS features [15]. Regarding aortic complications, overactivity of TGF-β enhances collagen production leading to reduced aortic wall compliance, upregulates the level of elastase and matrix-metalloproteinases (MMP) resulting in elastic fiber degradation, and this extracellular matrix remodeling eventually weakens the aortic wall making it more susceptible for aneurysm formation and dissection [15].

## Presentation, prevention and treatment of cardiovascular manifestations

The largest burden of MFS is associated with the cardiovascular involvement, aortic complications being the main cause of mortality [16]. The most severe cardiovascular manifestations are aortic root aneurysm and dissection, both of which can occur at other parts of the aorta as well. Apart from these, mitral valve prolapse with or without regurgitation, tricuspid valve prolapse or pulmonary artery dilation are also often seen in MFS [6]. Mitral valve prolapse is the leading cause of cardiovascular morbidity, mortality and cardiac surgery in children with severe MFS [2]. Of these cardiovascular features, aortic root dilation occurs most frequently, leading to aortic regurgitation and carrying the risk of aortic dissection and rupture [17].

Aortic dissection is a life-threatening manifestation and it develops at a significantly younger age in people with MFS compared to patients without MFS [18]. Approximately two-thirds of the cases are type A dissections [18], meaning that the ascending aorta is affected, requiring open surgical repair [19]. Type A dissection needs to be prevented by prophylactic aortic root replacement to provide the patients with a long-term survival accompanied by a good quality of life. Aortic root replacement has two main types. Bentall operation has been the gold standard method, involving the replacement of the aortic root with a mechanical valved conduit. Due to the implantation of a mechanical valve, its main disadvantage is the need for life-long anticoagulation and its associated complications [20]. This issue can be overcome by keeping the patient’s native valve with a valve sparing root replacement procedure. This comprises the remodeling technique also known as Yacoub operation and the reimplantation method also known as David surgery [21]. The main advantage of the remodeling is the preservation of aortic root function, while reimplantation stabilizes the annulus. These can be carried out for patients with aortic valves minimally affected by the disease [22]. In case of prophylactic indication, Bentall operations and valve sparing techniques seem to have similar long-term results [23].

Prophylactic aortic root replacement is associated with more favorable short- and long-term outcomes compared to aortic root replacement carried out due to acute aortic dissection. The most important difference can be found in the surgical mortality rates. A prophylactic aortic root replacement carries a risk of death of approximately 2%, while the operative mortality of an acute type A aortic dissection can be as high as 20%-25% [24–26]. In addition, emergency aortic root replacement also results in worse long term survival [27]. Furthermore, emergency procedures are less likely to be carried out by applying valve sparing techniques, leading to repeated aortic operations more often than elective surgeries. In the long term, MFS patients undergoing emergency aortic root replacement were more frequently presented with chronic dissection distal to the operation site and with larger aortic diameters throughout the entire aorta except for the abdominal part as well as with lower quality of life accompanied by a lower activity score [28]. Furthermore, MFS patients who underwent acute life-saving aortic root replacement showed a significantly higher trait anxiety level than the normal population, but this difference was not observed between patients with a prophylactic aortic root replacement and the normal population [29].

The obvious benefits of a prophylactic aortic root replacement highlight the pivotal role of a highly sensitive and specific risk stratification system to identify patients in need for a prophylactic intervention. Currently the indication criteria of a preventive surgery are centered around aortic diameter: in MFS an ascending aorta with the size of ≥ 50 mm is an indication for the procedure, while in case of any of the risk factors stated in the guidelines (family history of aortic dissection, desire for pregnancy, severe aortic- or mitral regurgitation, systemic hypertension and/or aortic size increase  > 3 mm/year) intervention should be considered at the size of ≥ 45 mm [30]. However, in a subset of MFS patients aortic dissection occurs before aortic diameter reaches the threshold required for the indication of prophylactic repair [28, 31, 32], making the aortic diameter less reliable as the main indicator for a preventive procedure [33]. On the other hand, having the threshold too low would result in exposing patients in whom a dissection would never develop to the risks of a cardiac surgery [34]. These findings demonstrate the need for the improvement of the current risk stratification system, to be able to optimize patient selection for the prophylactic aortic root replacement.

Great effort has been made to provide this system with the potential to improve, as data on various factors associated with aortic complications have been accumulating. Therefore, the aim of this review is to explore these possible predictors of severe aortic involvement in MFS patients, with a special attention paid to the current knowledge on the rapidly expanding genotype–phenotype correlations.

## Biomarkers

**Fig. 1:**
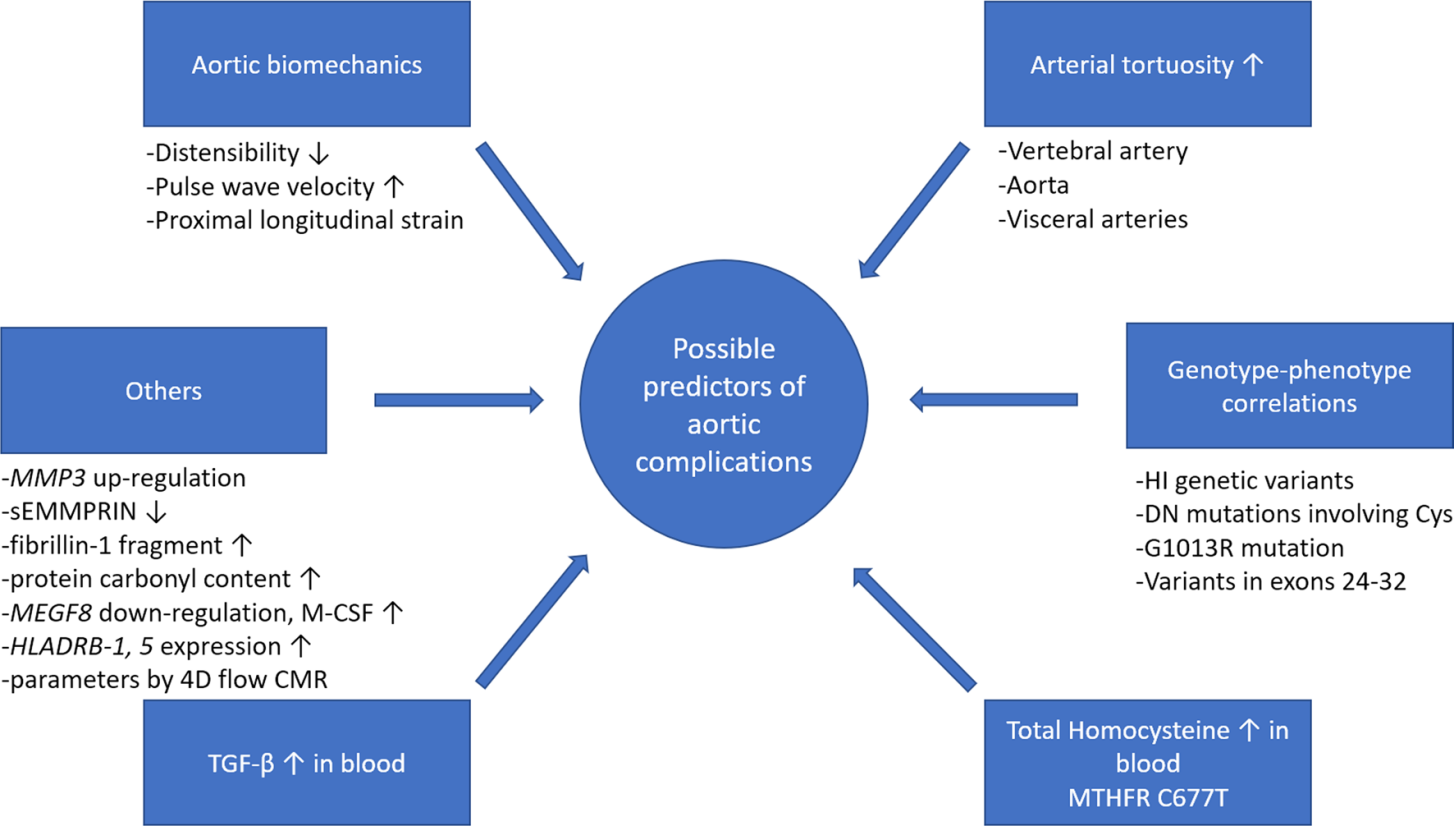
Possible predictors. This figure shows the discussed possible predictors of severe aortic involvement in Marfan syndrome based on the current knowledge of the field. These include biomarkers measured in blood like TGF-β and homocysteine, radiological biomarkers as arterial tortuosity and aortic biomechanics, genotype–phenotype correlations and some other potential predictors

Biomarkers are required to predict the development of severe aortic involvement, especially acute type A aortic dissection in MFS patients. Several studies with varying success have been carried out to identify the predictors of severe aortic manifestations. Figure 1 summarizes the potential biomarkers discussed in this review.

## Biomarkers measured in blood samples

### *TGF-*β

One of these biomarkers is TGF-β, which is a paracrine regulatory molecule with a role in several biological processes, and its elevated level is a key component in the development of the Marfanoid phenotype [15]. Based on the pathomechanism, a higher bioavailability of the active molecule should lead to more severe clinical features. Therefore, the level of TGF-β has been assessed as a possible predictor, although the results are inconsistent. Ogawa et al. investigated the total TGF-β1 in blood in 32 MFS patients and they did not find it to be elevated compared to the control group, and no correlations were identified between TGF-β1 concentration and phenotype severity [35]. However, in another study, blood total TGF-β1 was increased in patients with MFS compared to non-MFS individuals, but no significant correlation was shown with the size of sinuses of Valsalva and Z-score. Importantly, total TGF-β1 level was lower in MFS patients receiving losartan, beta blockers or both, in comparison to untreated individuals [36]. Similarly, when Franken and colleagues analyzed the level of TGF-β in blood in a larger cohort of 99 MFS patients, a significantly higher baseline blood TGF-β level was revealed in MFS patients compared to healthy controls. They also investigated the possible correlation of blood TGF-β level with progressive aortic root dilation and dissection, and they found that aortic root diameters correlated significantly with circulating TGF-β concentrations in MFS patients without previous aortic surgery. Moreover, patients with a prior aortic root replacement had significantly increased circulating TGF-β compared to patients without prior aortic operation. After a mean follow-up of 3 years, circulating TGF-β levels significantly correlated with aortic root growth. Patients with a TGF-β concentration above 140 pg/ml had a 6.5-fold-increased risk for the combined endpoints of aortic dissection and elective aortic root operation [37]. Our group also investigated the possible predictor role of TGF-β. We measured the level of active TGF-β in blood in 24 patients with MFS, and it appeared to be higher in MFS patients with prior surgery compared to the control group of non-MFS individuals, and in the aortic dissection group levels were higher than in patients with annuloaortic ectasia. TGF-β was revealed to be an independent predictor for aortic dissection [33]. Kim et al. also found elevated concentrations of active and latent TGF-β1 in peripheral blood of MFS patients compared to non-MFS individuals, and in contrast to the previously mentioned studies, beside the clinical outcomes Kim et al. sought to determine the correlation of blood TGF-β1 levels with the degree of cystic medial degeneration. The level of active TGF-β1 in blood positively correlated with SMAD2 phosphorylation (i.e. the activation of SMAD2) and with the severity of elastic fiber fragmentation, apoptosis and ground substance deposition in the medial layer of aneurysmal aortic tissue of MFS patients. SMAD2 phosphorylation level was also found to be correlated with the severity of pathological changes in the aortic wall. However, no association was found between blood TGF-β1 level and aortic root diameter, and no significant difference was observed in terms of the investigated features in patients with and without aortic dissection at the time of surgery. As Kim and colleagues state, this could be explained by the fact that MFS patients with severe aortic manifestations were included in the study. It is important to note that they included 41 MFS patients, but blood samples were collected only from 10 of them [38]. Circulating TGF-β levels were also evaluated in 28 non-MFS patients within the first 24 h of acute aortic dissection. They found fivefold elevations in the aortic dissection group compared to the control group, and the level of TGF-β was increased in type A dissections compared to type B ones, suggesting a possible biomarker role of circulating TGF-β even in non-MFS patients [39].

The different results of the described studies could be due to many factors. These include population size, disease severity of studied individuals, treatment with cardiovascular medications and also ex vivo platelet degranulation can influence the results [35]. Well-designed multicenter prospective studies involving large number of patients are required to decide on the role of TGF-β levels in the prediction of aortic complications, before it could be applied in the clinical management of MFS patients.

### Homocysteine

Homocysteine is a sulfhydryl amino acid and it is a key member of the methionine cycle. Elevated levels of blood homocysteine have been associated with vascular disease development [40], and homocystinuria is a related disorder of MFS characterized by Marfanoid habitus, ectopia lentis, mental retardation and thrombotic events [6].

The association of homocysteine levels and methylenetetrahydrofolate reductase (MTHFR) C677T polymorphism with the severity of cardiovascular involvement has been evaluated in MFS patients. Significantly increased blood total homocysteine (tHcy) levels were found in patients with aortic dilation and/or aortic dissection (the group with severe involvement) compared to individuals with mild cardiovascular manifestations. Furthermore, the aortic dissection subgroup was revealed to have highertHcy levels and a higher prevalence of C677T homozygotes than the severe group without aortic dissection, and patients with aortic dissection also had a larger prevalence of C677T homozygotes than the mild cardiovascular group. After multivariate regression analyzes, homocysteinemia was associated with the risk of severe cardiovascular involvement or aortic dissection [41]. Similarly, our group identified homocysteine blood level as an independent risk factor for severe cardiovascular involvement including aortic dissection in MFS. In this study, the level of homocysteine was significantly increased in individuals with aortic dissection compared to other groups of patients with or without mild cardiovascular manifestations, severe aortic dilation and controls. Folate blood level was also measured and it appeared to be significantly lower in the aortic dissection group than in the other ones. In the same study, four polymorphisms of three folic acid metabolism enzymes, namely MTHFR, 5-methyltetrahydrofolate-homocysteine methyltransferase (MTR) and 5-methyltetrahydrofolate-homocysteine methyltransferase reductase (MTRR) were analyzed, and used to calculate a single nucleotide polymorphism (SNP) risk score. This score was the highest in patients with aortic dissection compared to the other groups, meaning that the aortic dissection group had the largest prevalence for homozygotes and heterozygotes for the four investigated gene polymorphisms. A genotype–phenotype correlation was found between homocysteine blood level and the examined gene polymorphisms [42]. A possible explanation for the association of elevated homocysteine levels and cardiovascular involvement severity can be the high susceptibility of the key aortic wall component, fibrillin-1 to homocysteinylation due to its great number of EGF-like domain content. Homocysteinylation damages proteins with various mechanisms, one of which is the disruption of disulfide bridge formation [43, 44]. Therefore, increased levels of homocysteine could be used to predict the development of a more malignant aortic phenotype. Furthermore, vitamin B12 supplementation could reduce homocysteine level with the potential of lowering the risk of aortic complications [42].

## Biomarkers assessed by medical imaging

### Arterial tortuosity

A promising predictor of severe aortic involvement can be the assessment of arterial tortuosity. Arterial tortuosity is a feature of hereditary aortopathies, mainly characteristic not only for arterial tortuosity syndrome but also for Loeys-Dietz syndrome (LDS) as well as also present in MFS, aneurysm-osteoarthritis syndrome and familial thoracic aneurysms and aortic dissections. Arterial tortuosity can be defined by the increased number of turns on the involved artery [45]. Shaine A. Morris and colleagues hypothesized that increased arterial tortuosity could indicate vessel fragility and its objective measure could be associated with clinical outcome in connective tissue disorders. They analyzed magnetic resonance angiography images and calculated the vertebral tortuosity index (VTI) for 90 patients with confirmed connective tissue diseases including 57 individuals with MFS. They report that vertebral artery tortuosity is common in MFS, and regarding the connective tissue disorder group, a higher VTI was found to be associated with more severely dilated aortic root, higher cardiac surgery rate, and younger age at aortic dissection, heart surgery and death [46]. Franken et al. investigated 211 MFS patients with 3D MR imaging and they defined the aortic tortuosity index (ATI) with the aim to serve as a marker for aortic disease severity and a predictor of clinical outcome, like elective aortic surgery and aortic dissection. They found a lower ATI in controls compared to matched MFS patients. ATI showed a positive correlation with aortic diameter and aortic volume expansion rate in 3 years follow-up, but it did not correlate with aortic root dilation rate. ATI was found to be an independent predictor of clinical outcome, including type B aortic dissection. Type A aortic dissection did not occur during the follow-up period [47]. As the geometry of the examined vessels can be influenced by the skeletal features of MFS, our group previously decided to assess the tortuosity of visceral arteries that are less likely to be altered by the skeletal manifestations. We investigated the correlation between the tortuosity of visceral arteries (splenic artery, right and left renal artery) and the severity of cardiovascular involvement in 37 MFS patients, and additional geometric metrics were calculated to provide a more precise description of the analyzed arteries. We found that the tortuosity of the mentioned arteries was increased in MFS compared to controls, with the geometry being dominated by higher amplitude and lower frequency curves. Patients with prior aortic surgery had increased tortuosity in the right and left renal arteries in comparison with MFS patients without previous aortic operation, suggesting visceral artery tortuosity as a possible new predictor of severe aortic involvement in MFS [48].

The mechanism of the development of increased arterial tortuosity is not known, but there are studies that hypothesized the role of oxidative stress in arterial tortuosity syndrome [49], abnormal lengthening of the arteries probably caused by maladaptation to axial stress, resulting in curving and bending [45], and increased TGF-β activity [45, 46]. As discussed above, the latter can also be a potential predictor of severe aortic involvement.

In summary, there is a strong indication that increased tortuosity can be observed on multiple arterial segments in MFS patients, and the degree of tortuosity could be associated to the severity of aortic involvement. However, the latter association should be further investigated to determine its predictive value.

### Aortic biomechanics

Another field where potential biomarkers could arise is aortic biomechanics, mostly referring to aortic stiffness. Aortic distensibility and pulse wave velocity (PWV), which are markers for aortic stiffness, were measured at ascending-, descending- and abdominal aortic segment levels using MRI in 80 MFS patients without advanced aortic disease and in 36 age- and sex-matched controls. In the MFS group aortic distensibility was lower at the investigated three aortic levels, and aortic arch-, and ascending-to-abdominal aorta PWV appeared to be higher than in the control group. MFS patients with and without aortic root dilation were both found to have lower aortic distensibility and increased aortic arch PWV than controls at the investigated three levels, but ascending-to-abdominal aorta PWV was only higher in the group with dilated aorta. Therefore, this study showed that aortic stiffening is present in MFS patients without advanced aortic involvement [50]. These findings could indicate that aortic involvement occurs before dilation, however, longitudinal studies are required to evaluate their predictive value on severe aortic manifestations. A study with a median follow-up of 2.7 years involving patients with connective tissue disorder, mainly MFS, found that lower aortic distensibility was independently associated with aortic root replacement surgery, and higher rates of aortic dilation were present in case of lower ascending aortic strain. The authors also showed that higher aortic stiffness was associated with higher vertebral tortuosity index, the previously discussed potential predictor for aortic complications [51]. Among other findings, Nollen et al. identified that a decreased distensibility of the descending thoracic aorta had an independent predictive value for the progressive dilation of the descending thoracic aorta [52]. Mortensen et al. applied applanation tonometry to assess aortic stiffness and pulse wave reflection and their correlation with aortic disease severity in 50 MFS patients for a mean follow-up time of 22 months. Augmentation index was found to be associated with aortic disease progression, while lower aortic disease progression was demonstrated in case of lower augmentation index and lower PWV [53]. When investigating the use of regional PWV in the prediction of regional aortic luminal growth for a 2 year period, more than 78% of MFS patients without regional aortic luminal growth was found to have a normal PWV value at baseline [54]. This finding could serve as a negative predictor of progressive aortic disease. In a recently published prospective study involving 117 MFS patients followed-up on an average of 85.7 months, proximal aorta longitudinal strain was identified as an independent predictor of aortic dilation, and it was independently related to the fastest aortic dilation and to aortic events (aortic dissection and elective surgery) [55].

These findings together suggest that apart from aortic root size, other parameters assessed by medical imaging could be considered in the risk evaluation for aortic events in individuals with MFS.

Furthermore, the predictive role of previous aortic root or ascending aortic surgery for the occurrence of type B aortic dissection should be also highlighted here [56]. Diameters of the distal aorta were also found to be larger in patients with than without previous aortic surgery. One explanation can be that patients requiring surgery are more severely affected by the disease, however, it is also important to consider that intervention on the proximal aorta can have an impact on the distal parts due to hemodynamic and wall mechanics alterations [57]. These findings necessitate a close monitoring of the distal aorta after operations of the proximal segments.

## Genotype–phenotype correlations

An expanding and promising field that aims to improve the risk stratification of severe aortic events in MFS is the examination of possible genotype–phenotype correlations. The use of genetic background in the risk assessment, and in some cases even in clinical management has been already applied in cardiovascular diseases [58]. As an example, prophylactic aortic surgery in LDS can be considered at lower aortic diameter in the presence of *TGFBR2* mutation [30]. Genetic variants leading to an increased risk of aortic events could also be considered as biomarkers with the potential to contribute to the establishment of a more accurate risk stratification system for aortic dissection.

### Established associations

Despite the associations between phenotype and genetic background have been investigated since *FBN1* was identified as the causative gene for MFS [59], to date only a few widely accepted correlations exist.

One is in regard with the ocular system. Ectopia lentis, a characteristic feature of MFS, was found to be less frequent in PTC variants than in missense mutations, especially the ones with cysteine involvement, and this finding has been reported throughout the literature [60–63].

Other well-defined correlation is based on the location of the genetic variant. Mutations in exons 24–32 are associated with neonatal MFS, which is the most severe form of the disease with a short life expectancy. The main cause of death is congestive heart failure caused by mitral and tricuspid regurgitation in this patient population [64]. However, variants in this region have also been reported to result in atypically severe and classic MFS [65].

Furthermore, the *FBN1* mutation p.Gly1013Arg, which is located in the neonatal region (Exon 24) has been reported in a couple of unrelated patients with MFS, each time leading to atypically severe disease with advanced cardiovascular features and with a longer survival than the neonatal form [65–67].

### Aortic involvement severity based on variant type

Results have been conflicting in the field of genotype–phenotype correlations, which could be down to small sample sizes and differences in study designs or due to co-occurring genetic modifier(s) [1] or differences in blood pressure load damaging the aortic wall. However, certain trends have emerged that could give base for larger studies with the aim of establishing well-defined correlations that could contribute to the improvement of risk stratification of aortic complications in MFS patients.

Faivre et al. carried out their research on a large sample scale involving 803 probands with confirmed *FBN1* mutations and available clinical information based on the UMD-FBN1 database [68]. Regarding the cardiovascular features, they found that missense mutations eliminating a cysteine had a higher probability of ascending aortic dilation and mitral valve prolapse than mutations creating a cysteine. When comparing patients with a variant in the neonatal region (exons 24–32) to patients with mutations outside this region, the former group possessed a higher cumulative probability of ascending aortic dilation and aortic surgery before or at the age of 40 years. However, they did not find differences between the certain mutation types in terms of aortic complications [61].

Further correlations were revealed in a study involving 179 patients with confirmed *FBN1* mutations. In patients with aortic events (aortic dissection and/or surgery), the mutation type was mainly truncating (nonsense and frameshift) or splicing, only 21% were missense ones. Furthermore, truncating or splicing variants were present in only 39% of all probands without aortic event, and their frequency was also lower in Ghent-positive patients without aortic event. A trend was observed toward aortic dissection or surgery occurring at younger age in patients with a truncating or splicing mutation compared to people with a missense variant. A further trend was noted toward aortic dissection patients having truncating or splicing variants more frequently than patients who underwent a prophylactic surgery [69]. Further studies found that aortic dissection was more common in PTC variants than in mutations with cysteine substitution, however this difference was not significant [60, 70]. In contrast, Loeys et al. did not find a higher prevalence of aortic dissection in PTC mutations compared to cysteine substitutions [71], and cardiovascular manifestations did not differ among these 2 variant groups in other studies [72, 73]. Pees et al. did not reveal any difference in terms of aortic involvement when PTC and missense mutations were compared [74]. Wang et al. analyzed the associations in 39 *FBN1*-positive Chinese patients, and identified a higher probability of cardiovascular complications in case of PTC or splicing variants compared to missense ones. Interestingly, they report a more vulnerable cardiovascular system in patients with missense mutation not affecting a cysteine, than in case of cysteine affecting genetic variants [75].

Franken et al. classified *FBN1* mutations according to their effect on protein level as dominant negative (DN) or haploinsufficient (HI) variants [76]. In a simplified form, DN variants (e.g. missense mutations and in-frame insertions/deletions) lead to proteins with altered structure/function but with normal expression level. In contrast, HI mutations (PTC-introducing nonsense mutations and frameshift insertions/deletions) result in a reduced expression of the mutant allele and thus to reduced total amount of fibrillin-1. Franken and colleagues used the Dutch CONgenital CORvitia (CONCOR) registry database involving 570 MFS patients to assess the correlations between aortic complications and the HI and DN mutation types. Genetic testing was carried out for 433 patients and 357 pathogenic *FBN1* mutations were identified. Patients with a HI mutation reached the clinical endpoints of aortic dissection and aortic surgery significantly more frequently than individuals with DN variants. After a mean follow-up of about 8 years, the HI group had an increased risk for cardiovascular death (2.5-fold), for the combined clinical endpoint of cardiovascular death and aortic dissection (2.4-fold), and for any cardiovascular events (1.6-fold) than the DN group. Similarly, when investigating aortic events in patients without cardiovascular complications at the time of inclusion in the CONCOR registry, HI patients showed an increased risk for the combined endpoint of cardiovascular death and aortic dissection and for any aortic event. Age at aortic dissection was significantly lower for the HI mutations compared to the DN ones. In conclusion, the findings of Franken et al. suggest that HI genetic variants lead to more severe cardiovascular manifestations in MFS patients [77].

In another publication the authors have further investigated the effect of these mutations by assessing the aortic dilation rate and cardiovascular events among the HI and DN genetic variants. Having no difference in age and body surface area (BSA), patients with HI mutations were found to have larger aortic root diameter than the ones with a DN variant at baseline, but no difference existed in terms of aortic surgery and aortic dissection. However, the HI group underwent surgery at significantly younger age. The aortic dilation rate was significantly higher in the HI group at the level of the aortic root and tubular ascending aorta, but the other parts of the aorta did not differ in terms of dilation rate between the groups. In addition, HI tended to pose a higher risk of aortic dissection and cardiovascular death, compared to DN genetic variants [63].

Consistently with these results, patients with truncating mutations (nonsense or frameshift) experienced a higher rate of aortic events (aortic dilation over surgical thresholds and type A aortic dissection) and these events even—although not significantly—occurred at a younger age compared to missense variants in a study involving 90 patients with *FBN1* mutation [62]. Similarly, nonsense and frameshift mutations were found to be more deleterious in a cohort of 180 patients with confirmed *FBN1* mutation, as they were more frequent in patients with aortic dissection than in individuals with aortic aneurysm. On the contrary, the proportion of missense variants was higher in the aortic aneurysm than in the aortic dissection group. The authors also investigated the aortic wall specimens and found that nonsense and frameshift variants caused more severe pathological changes in the aortic wall than missense mutations [78]. In contrast, Hernándiz et al. did not identify significant difference in aortic involvement between HI and DN mutations in their study on 61 MFS patients. However, there was a tendency toward more frequent aortic involvement in missense mutations substituting a cysteine compared to other missense variants. Furthermore, the DN group had nearly significantly more cases without aortic involvement than the HI group. More patients with HI variant had aortic dissection and these occurred at younger age than in individuals with DN mutations, but the difference did not reach a statistically significant level [79].

Based on the results of the reviewed studies, it could be concluded that HI variants are more likely to result in a severe aortic phenotype. However, patients with DN mutations also experience aortic events, raising the question whether subtypes leading to severe cardiovascular manifestations could be identified within this group. This issue is also emphasized by the previously mentioned finding that mutations eliminating a cysteine lead to aortic dilation more frequently than the ones introducing this amino acid [61]. This observation demonstrates that not all missense mutations carry the same risk of aortic events, and also raises the possibility that variants eliminating cysteine has the potential to cause malignant cardiovascular phenotype, which could be explained by the important role of this amino acid in the protein structure reached by enabling disulfide bond formations. Indeed, these questions were considered in a study conducted by Takeda et al. involving 248 patients with *FBN1* mutations, where HI variants were again proved to have a higher risk for severe aortic events (type A aortic dissection, aortic root replacement and aorta-related death) than DN mutations, although they identified a subgroup within the DN variants with similar deleterious effect as HI ones. This subgroup comprised of patients with mutations affecting existing or creating new cysteine residues and in-frame deletions in the calcium-binding EGF (cb-EGF) domains of exons 25–36 and 43–49 (DN-CD). The newly identified DN-CD subtype had a significantly higher risk of severe aortic events than the remaining DN variants (DN-nonCD), but no significant difference was observed when comparing DN-CD and HI mutations. In addition, the DN-CD + HI group had a higher Z-score compared to DN-nonCD variants [8]. Similarly, our research group also further classified the DN variants based on their cardiovascular effect. The classification was influenced by the above-mentioned finding that elimination of a cysteine is more deleterious than the introduction of this amino acid. Therefore, we classified DN mutations into ones that eliminate a disulfide-bonding cysteine (DN Cys) and the ones not eliminating a disulfide-bonding cysteine (DN non-Cys). Comparing these two groups of DN variants, DN Cys was revealed to lead to aortic involvement (aortic dilation and/or dissection) more frequently than DN non-Cys variants. To make a classification system applicable within clinical settings, we created the combined group of the high-risk HI and DN Cys variants and compared it to DN non-Cys mutations. We found that aortic involvement was more frequent in the combined group of HI and DN Cys than in DN non-Cys. In addition, patients with DN Cys variants required aortic surgery more frequently than patients with HI and DN non-Cys mutations. Therefore, in our study DN Cys mutations appeared to be more deleterious than HI ones [80]. Similarly, when genotype–phenotype correlations were assessed in a pediatric cohort, missense variants affecting a cysteine showed a higher rate of sinuses of Valsalva dilation than missense mutations not affecting cysteine. However, no differences in cardiovascular involvement were observed when comparing the other mutation types [81].

Arnaud et al. investigated genotype–phenotype correlations in the cardiovascular and other systems in a large cohort comprising 1575 MFS patients with (likely) pathogenic *FBN1* variants. PTC mutations (nonsense, splice site and insertions/deletions of a number of nucleotides not divisible by 3) were found to lead to a more severe aortic phenotype, including a higher risk for aortic dissection or surgery, larger aortic root diameter despite younger age and a shorter life expectancy compared to in-frame variants (missense and small insertions/deletions of a number of nucleotides that are a multiple of 3). Arnaud et al. further categorized the in-frame variants according to cysteine involvement, creating the following groups: substitution of cysteine for another amino acid (-Cys), substitution for a cysteine (+ Cys) and missense variants not modifying cysteine (noCys). In-frame -Cys mutations were associated with severe cardiovascular features, + Cys variants resulted in fewer aortic events and the noCys group demonstrated an intermedier aortic risk. Notably, in-frame, especially -Cys variants in the region of exons 24–32 were associated with severe aortic involvement, while PTC variants in this region did not result in severe aortic phenotype [82].

Table 1 summarizes the main findings of the discussed articles on genotype–phenotype correlations.

**Table 1:** Genotype–phenotype correlations reported in the literature until the end of 2020

Authors	Year of publication	Number of patients compared	Compared genetic variants
A: No difference in terms aortic severity			
Loeys et al. [71]	2004	85	*PTC and Cys substitutions*
Arbustini et al. [73]	2005	81	*PTC and missense Cys*
Comeglio et al. [72]	2007	174	*PTC and missense Cys*
Faivre et al. [61]	2007	803	*all mutations apart from Cys elimination and Cys introduction*
Pees et al. [74]	2014	49	*PTC and missense*
B: Non-significant difference found in aortic severity			
Schrijver et al. [60]	2002	104	*PTC* > *Cys substitution*
Rommel et al. [70]	2005	76	*PTC* > *Cys substitution*
Hernándiz et al. [79]	2020	61	*DN Cys* > *DN non-Cys* *HI* > *DN*
C: Significant difference found in aortic severity			
Faivre et al. [61]	2007	803	*missense eliminating Cys* > *missense introducing Cys*
Wang et al. [75]	2013	39	*PTC or splicing* > *missense* *missense non-Cys* > *missense Cys*
Baudhuin et al. [69]	2015	179	*truncating or splicing* > *missense*
Franken et al. [77]	2016	357	*HI* > *DN*
Franken et al. [63]	2017	290	*HI* > *DN*
Becerra-muñoz et al. [62]	2018	90	*Truncating* > *missense*
Takeda et al. [8]	2018	248	*HI* > *DN* *DN-CD* + *HI* > *DN-nonCD*
Stark et al. [81]	2020	105	*missense Cys* > *missense non-Cys*
Stengl et al. [80]	2020	78	*DN Cys* > *DN non-Cys* *DN Cys* + *HI* > *DN non-Cys* *DN Cys* > *HI*
Xu et al. [78]	2020	180	*frameshift and nonsense* > *missense*
Arnaud et al. [82]	2021	1575	*PTC* > *in-frame* *-Cys* > *other missense*

The table presents the articles published so far on genotype–phenotype correlations in 3 groups according to the level of difference in aortic manifestation severity between the mutation types (A: no difference, B: non-significant difference, C: significant difference). The greater-than sign between the mutation types indicates the ones with the more severe aortic involvement. The detailed differences can be found in the text

### Future perspectives for genotype–phenotype correlations

**Fig. 2:**
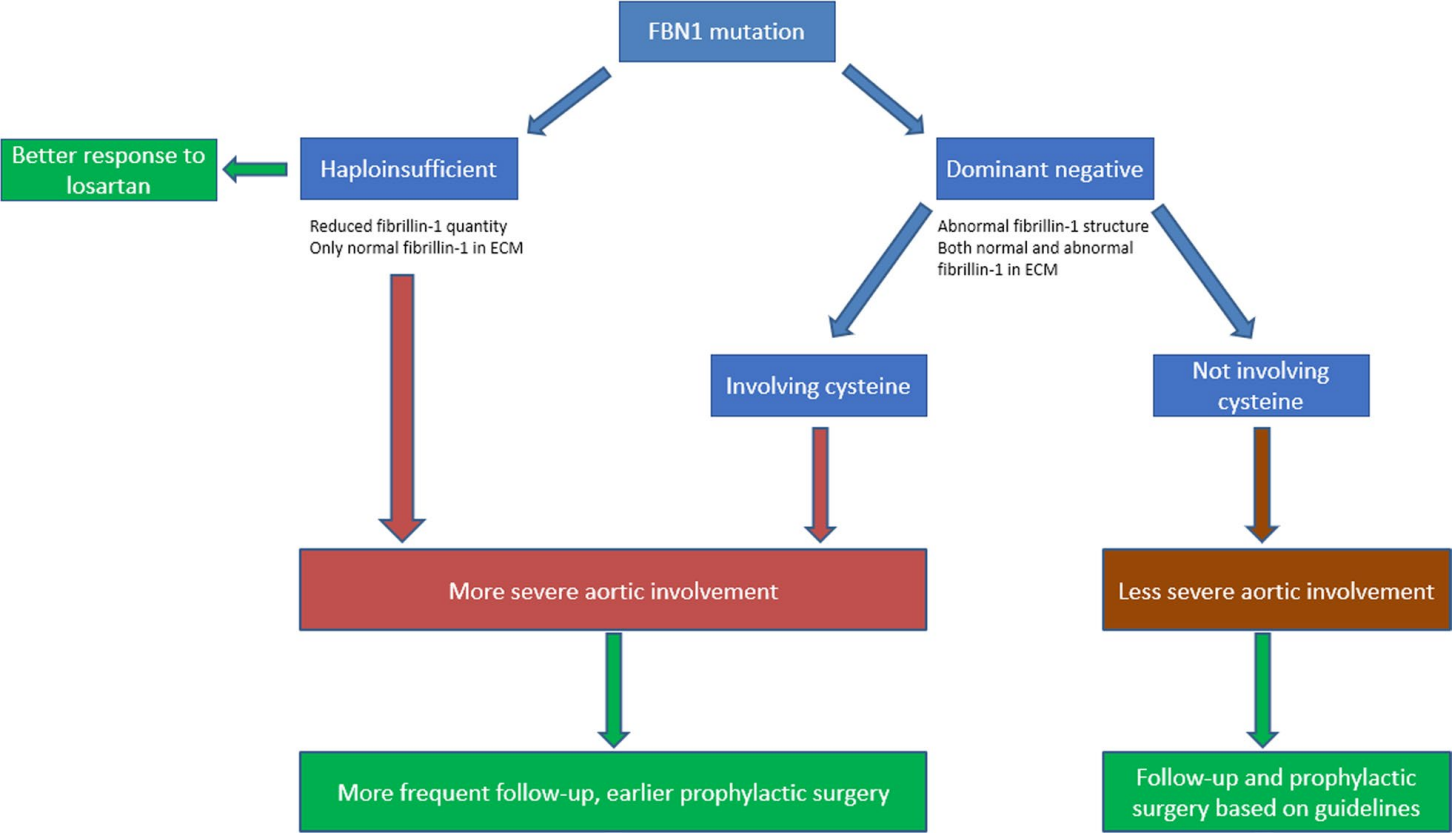
Proposed clinical management strategy of aortic involvement in Marfan syndrome based on the mutation type. This figure demonstrates a proposed management strategy in MFS patients based on their mutation type, according to the recent findings of genotype–phenotype correlation studies. Based on the results of the reviewed articles, HI and DN Cys variants seem to carry a higher risk for aortic complications than DN non-Cys mutations, therefore they require a more frequent patient follow-up and an earlier prophylactic procedure than DN non-Cys variants. This approach needs to be confirmed by larger, prospective studies before it can be applied within clinical settings. In the figure, blue boxes represent the type of genetic variants, the color red indicates a more severe, while brown shows a less severe aortic involvement. The green boxes demonstrate the proposed management approach.

Based on the results so far, considering the genetic background of a patient could contribute to a more accurate risk stratification for severe cardiovascular involvement. Figure 2 presents a potential management approach in MFS patients based on their genetic background. According to this, HI and DN Cys variants are more likely to lead to more severe aortic involvement, than DN non-Cys mutations, therefore patients with HI and DN Cys mutations require more frequent follow-up and earlier prophylactic aortic root replacement than individuals with DN non-Cys mutations. In case of the latter group, current guidelines could be applied. This approach has been proposed in our previous work for patients with MFS [80], and the above reviewed results seem to strengthen this potential management strategy.

However, before clinical application, further investigations are in need with larger patient cohorts and in a prospective manner in order to precisely estimate the risk of the certain mutation types.

Besides their prognostic value, genotype–phenotype associations could also play a role in the decision making of medication selection. Losartan, an angiotensin II type 1 receptor (AT1) antagonist showed more promising results in the correction of aortic complications in a mouse model of MFS compared to beta-blockers [83], however, no difference was found between losartan and atenolol in the rate of aortic root dilation when investigated in humans [84]. In addition to pharmacogenetic predisposition to the metabolization of losartan [85], genotype–phenotype correlations could provide a solution for this issue, as Franken et al. identified MFS patients with HI mutation to have a better response to losartan therapy in terms of reducing aortic dilation rate compared to patients with DN variant [76]. This highlights the potential use of genetic background in medication prescription for MFS patients to provide more personalized therapy. This is also demonstrated in Fig. 2.

Genotype–phenotype correlations have the possibility to be used in a wide-range of areas, further studies are required to unveil their full potential to reach personalized patient care.

The GeneReviews article on MFS by H. Dietz, which covers all the relevant aspects of the syndrome, states that there are no definitive genotype–phenotype correlations, therefore identifying the pathogenic variant of the patient carries no real prognostic and therapeutic value [2]. However, as we demonstrated, promising information on this issue has been growing, therefore in the future it may be advisable to rethink the role of these associations in patient care.

## Other potential biomarkers

Besides the discussed potential biomarkers, other factors have also been investigated as possible predictors of serious cardiovascular manifestations in MFS. In our above-mentioned study about increased TGF-β levels as a promising biomarker, we assessed further possible predictors. We found that the *MMP3* gene in peripheral blood mononuclear cells showed significant up-regulation in patients with dissection in comparison with the annuloaortic- and control groups, and also striae atrophicae was significantly more common in patients with aortic dissection than in patients with annuloaortic ectasia [33]. MMPs are upregulated by TGF-β in the aortic wall and they cause elastic fiber degradation [86]. Based on the important role of MMPs in aortic disease pathogenesis in MFS, the blood level of soluble form of extracellular MMP inducer (EMMPRIN) that has already been investigated in several diseases was analyzed in 42 MFS patients. It was found that soluble EMMPRIN levels in blood negatively correlated with the severity of aortic dilation according to the Z-score. Patients with aortic ectasia defined by Z-score ≥ 2 had lower soluble EMMPRIN levels than patients without aortic ectasia [87]. Another potential biomarker originating from extracellular matrix degradation is blood fibrillin fragment concentration. Marshall et al. measured the level of fibrillin-1, fibrillin-2 and fibulin-4 fragments in blood samples of 1265 patients with aortic aneurysm and dissection, including individuals with MFS. They found that fibrillin-1 and fibrillin-2 fragments were detectable in a higher proportion of patients with aneurysm than in controls, while fibulin-4 was detectable more frequently in control samples. In addition, patients with thoracic aortic aneurysm who developed acute or subacute aortic dissection were more likely to have higher fibrillin-1 fragment levels than thoracic aortic aneurysm patients without dissection, indicating a more severe aortic involvement [88].

Fiorillo et al. examined the levels of the oxidative stress marker, protein carbonyl content (protein CO) in blood in 32 MFS patients. Protein CO appeared to be higher in the blood of individuals with MFS compared to the control group, and it was also elevated in patients with major cardiovascular and skeletal, or major cardiovascular and ocular involvement in comparison to the remaining patients [89].

When investigating gene expressions in 55 MFS patients, multiple EGF like domains 8 gene (*MEGF8)* was significantly down-regulated in patients with aortic root dilation compared to the non-dilated group. Furthermore, the expression of major histocompatibility complex, class II, DR beta 1 and 5 genes (*HLADRB-1* and *HLADRB-5)* was significantly increased in the progressive dilation group in comparison to patients with low dilatation rate. This finding indicates a role of increased inflammation in severe aortic involvement. The authors also measured the levels of TGF-β and inflammatory markers in blood. TGF-β was elevated in patients with aortic dilation compared to patients with normal sized aortic root, but after correction for sex, age and BSA, TGF-β levels did not correlate with aortic root diameter nor with the progression of aortic root size. However, one inflammatory cytokine, namely Macrophage-Colony Stimulating Factor (M-CSF) was found to correlate with the progression of aortic root dilation [90].

A recently emerging, promising tool to identify potential hemodynamic and biomechanical predictors is 4D flow cardiac magnetic resonance (CMR) imaging. Guala et al. used this technique to analyze blood flow and wall shear stress (WSS) patterns in 75 MFS patients, including 20 without aortic dilation and 48 healthy controls. Among other findings, the authors identified reduced in-plane rotational flow in the distal ascending and proximal descending aortic regions in MFS patients even in the absence of dilation, and also revealed a statistically significant reduction of circumferential WSS in the left/inner regions of the proximal descending aorta in MFS without dilation. The authors suggest that in-plane rotational flow and circumferential WSS could serve as an early marker of descending aortic dilation in MFS [91]. In a longitudinal evaluation of aortic hemodynamics in 19 adolescent MFS patients with 3.5-year follow-up, hemodynamic parameters remained stable over time. Compared to healthy controls, however, a significant reduction in regional WSS of the inner segment of the proximal descending aorta associated with abnormal localized flow patterns and enlarged diameter was detected. These alterations became more pronounced during the follow-up, but no significant changes were observed in other aortic segments, only in the proximal descending aorta [92]. Further studies have also been carried out to identify hemodynamic and biomechanical patterns with 4D flow CMR in MFS patients with the aim of detecting clinically relevant predictors for aortic manifestations [93, 94].

The factors demonstrated in this last section of biomarkers have been only sporadically reported, but further investigations could determine their role as possible predictors and their relation to the already identified predictors.

**Fig. 3:**
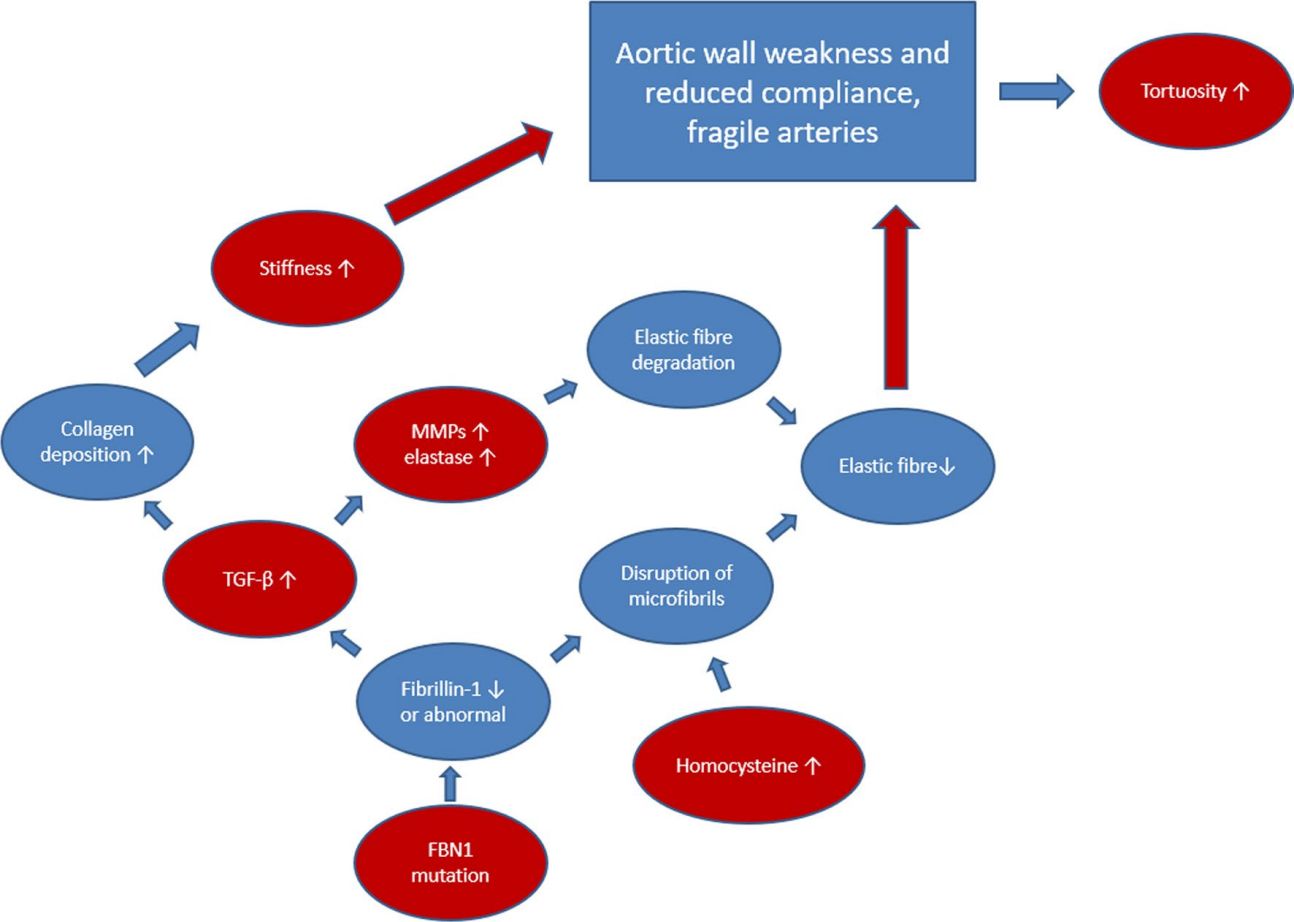
Pathomechanism of the manifestations of Marfan syndrome and connection points of the potential predictors. Our current understanding of the pathomechanism of aortic complications in Marfan syndrome is demonstrated in this figure, also highlighting those alterations that were previously described as possible predictors for severe aortic complications. The red figures illustrate the biomarkers discussed in this review, while the blue ones demonstrate the other relevant aspects in the pathomechanism of the manifestations of Marfan syndrome. The red arrows show the final causal steps in the formation of aortic complications, and the blue ones indicate the connections between the processes taking part in the pathomechanism

Figure 3 demonstrates the main constituents of the pathomechanism of the aortic involvement in MFS highlighting connection points of the discussed predictors.

## Conclusions

MFS patients face an increased risk of aortic complications that greatly contribute to the overall mortality and morbidity of the disease. Several factors were demonstrated so far to correlate with the severity of aortic manifestations and these could be used to improve risk stratification, to optimize patient follow-up and the indication criteria for a preventive surgery. These possible predictors play a role in the pathogenesis or are the consequence of the disease and they can be assessed by laboratory and imaging studies. The main identified biomarkers are circulating TGF-β and homocysteine levels, arterial tortuosity, aortic biomechanical parameters and genotype–phenotype correlations. Larger, unifying studies are required to further evaluate the role of these findings to be able to combine them with the systolic blood pressure load as the main trigger of aortic dissection and rupture, creating a more accurate risk stratification system for MFS patients.

## Data Availability

Not applicable.

## Abbreviations

ATI: Aortic tortuosity index
AT1: Angiotensin II type 1 receptor
BSA: Body surface area
cb-EGF: Calcium-binding EGF
CNV: Copy number variation
CONCOR: CONgenital CORvitia
DN: Dominant negative
DN-CD: DN variant affecting or creating cysteine residues and in-frame variants in the central cb-EGF domains (exons 25–36 and 43–49)
DN-Cys: DN mutation eliminating a disulfide-bonding cysteine
DN-nonCD: DN variant neither affecting nor creating cysteine residues and in-frame variants in the central cb-EGF domains (exons 25–36 and 43–49)
DN non-Cys: DN mutations not eliminating a cysteine
EGF: Epidermal growth factor
EMMPRIN: Extracellular MMP inducer
*FBN1*: Fibrillin-1 gene
HI: Haploinsufficient
*HLADRB-1*: Major histocompatibility complex, class II, DR beta 1 gene
*HLADRB-5*: Major histocompatibility complex, class II, DR beta 5 gene
LAP: Latency associated peptide
LDS: Loeys-Dietz syndrome
LLC: Large latent complex
LTBP: Latent TGF-β binding protein
*MEGF8*: Multiple EGF like domains 8 gene
M-CSF: Macrophage-colony stimulating factor
MFS: Marfan syndrome
MMP: Matrix metalloproteinase
MTHFR: Methylenetetrahydrofolate reductase
MTR: 5-Methyltetrahydrofolate-homocysteine methyltransferase
MTRR: 5-Methyltetrahydrofolate-homocysteine methyltransferase reductase
noCys: Missense variants not modifying cysteine
protein CO: Protein carbonyl content
PTC: Premature termination codon
PWV: Pulse wave velocity
SLC: Small latent complex
SNP: Single nucleotide polymorphism
TB: TGF-β-binding protein-like
TGF-β: Transforming growth factor-β
TGFBR2: Transforming growth factor beta receptor 2 gene
tHcy: Total homocysteine
VTI: Vertebral tortuosity index
WSS: Wall shear stress
+Cys: Missense variant substituting another amino acid for a cysteine
−Cys: Missense variant substituting a cysteine for another amino acid
